# Bioaugmentation of PAH-Contaminated Soils With Novel Specific Degrader Strains Isolated From a Contaminated Industrial Site. Effect of Hydroxypropyl-β-Cyclodextrin as PAH Bioavailability Enhancer

**DOI:** 10.3389/fmicb.2019.02588

**Published:** 2019-11-14

**Authors:** Jaime Villaverde, Leonila Láiz, Alba Lara-Moreno, J. L. González-Pimentel, Esmeralda Morillo

**Affiliations:** Instituto de Recursos Naturales y Agrobiología de Sevilla (IRNAS-CSIC), Seville, Spain

**Keywords:** soil, bioaugmentation, cyclodextrin, *Stenotrophomonas maltophilia*, *Achromobacter xylosoxidans*, PAHs

## Abstract

A PAHs-contaminated industrial soil was analyzed using PCR amplification of the gene 16S ribosomal RNA for the detection and identification of different isolated bacterial strains potentially capable of degrading PAHs. Novel degrader strains were isolated and identified as *Achromobacter xylosoxidans* 2BC8 and *Stenotrophomonas maltophilia* JR62, which were able to degrade PYR in solution, achieving a mineralization rate of about 1% day^–1^. *A. xylosoxidans* was also able to mineralize PYR in slurry systems using three selected soils, and the total extent of mineralization (once a plateau was reached) increased 4.5, 21, and 57.5% for soils LT, TM and CR, respectively, regarding the mineralization observed in the absence of the bacterial degrader. Soil TM contaminated with PYR was aged for 80 days and total extent of mineralization was reduced (from 46 to 35% after 180 days), and the acclimation period increased (from 49 to 79 days). Hydroxypropyl-ß-cyclodextrin (HPBCD) was used as a bioavailability enhancer of PYR in this aged soil, provoking a significant decrease in the acclimation period (from 79 to 54 days) due to an increase in PYR bioavailable fraction just from the beginning of the assay. However, a similar global extension of mineralization was obtained. *A. xylosoxidans* was then added together with HPBCD to this aged TM soil contaminated with PYR, and the total extent of mineralization decreased to 25% after 180 days, possibly due to the competitive effect of endogenous microbiota and the higher concentration of PYR in the soil solution provoked by the addition of HPBCD, which could have a toxic effect on the *A. xylosoxidans* strain.

## Introduction

Organic pollutants released by the increasing number of industries is the direct cause of environmental impacts that have detrimental effects on living beings (Paul et al., 2005). Polycyclic aromatic hydrocarbons (PAHs) are pervasive hydrophobic organic compounds, which consist of two or more aromatic rings. Under natural environmental conditions, PAHs are not easily dissipated. Persistence increases with an increase in the molecular weight (Agrawal and Shahi, 2017). Low bioavailability and high persistence are important properties that make PAHs a cause for concern (Badr et al., 2004; Zhao et al., 2017), along with the potential toxic, mutagenic and carcinogenic effects of these compounds. PAHs are listed as priority pollutants by the United States Environmental Protection Agency (U. S. Environmental Protection Agency, 2008).

Bioremediation is considered as an useful and eco-friendly technology for cleaning up polluted environments using living organisms (Isaac et al., 2017). In natural soil bioremediation, the existing native microflora already present in the polluted soil is used to degrade the target contaminants (bioattenuation). Microorganisms that can degrade organic pollutants have been isolated with the aim of using their metabolic potential for the remediation of polluted soils (Paul et al., 2005). Especially, hydrocarbons have been part of the biosphere for millions of years, and also, a diverse group of eukaryotes has evolved to degrade them. From this group, most of the cultures are fungi, but there are also examples from several algal phyla, and there are reports that some protozoa can degrade hydrocarbons (Prince, 2018). Spini et al. (2018) evaluated the evolution of bacterial and fungal communities enriched from polluted soil by culture independent and dependent methods, concluding that the strains isolated reflected the microbial composition of the enriched consortia. The use of pure degrader strains is a recommended way to seek metabolic pathways or to evaluate the effect of different environmental conditions on PAHs biodegradation. A disadvantage of this approach is that it does not take into account the possibility that organic pollutant biodegradation can be the result of a synergic process between different microbial strains (Molina et al., 2009).

Bioaugmentation is defined as a technique for improvement of the degradative capacity of contaminated areas by the introduction of specific competent strains or consortia of microorganisms (Mrozik and Piotrowska-Seget, 2010). Bioaugmentation has been proven successful in the cleaning up of sites contaminated with aromatic compounds but still faces many environmental problems (Kauppi et al., 2011). One of the most difficult issues is survival of strains introduced to soil. It has been observed that the number of exogenous microorganisms has decreased shortly after soil inoculation. Many studies have shown that both abiotic and biotic factors influence the effectiveness of bioaugmentation (Cycon et al., 2017). This contaminant removal technique should be applied when the natural attenuation and biostimulation have failed (Mrozik and Piotrowska-Seget, 2010). In fact, bioaugmentation seems to be effective for the removal of PAH compounds at contaminated sites (Wu et al., 2016) or for the remediation of pesticides and their residues from soil (Cycon et al., 2017). In terms of efficiency and economy, this strategy for treating contaminated sites gives better results than chemical and/or physical methods (Isaac et al., 2017).

Numerous microorganisms have been isolated that are capable to degrade PAHs, but less are able to biodegrade chemical structures with four or more aromatic rings (Bastiaens et al., 2000). It is essential to be sure that these microorganisms do not produce more toxic metabolites than the pollutant parent compound during PAH degradation. *Stenotrophomonas maltophilia* strain VUN 10,003 was isolated by enrichment technique and evaluated for four and five PAHs aromatic rings in a basal liquid medium for fluorene (FLU) degradation and co-metabolization of other PAHs (Juhasz et al., 2000). Nzila et al. (2018) isolated a novel strain capable of metabolizing the four fused aromatic rings, identified as *Achormobacter xylosoxidans* PY4, which was able to utilize PYR in solution as the sole source of carbon, degrading more than 50% of the PYR in solution.

The low bioavailability of PAHs in soil affects their biodegradability, as they are considered hydrophobic compounds, and therefore, they sorb strongly to soil organic matter (Cerniglia, 1993). Cyclodextrins (CDs) are compounds capable of increasing water solubility of hydrophobic compounds. Organic compounds of the appropriate shape and size can form inclusion complexes with the low-polarity cavity of CDs, including PAHs (Morillo et al., 2012; Sánchez-Trujillo et al., 2013), pesticides and nitroaromatic compounds, provoking an increase of the bioavailability of these pollutants for degradation (Villaverde et al., 2013; Morillo et al., 2014; Morillo and Villaverde, 2017). Over organic solvents and/or non-ionic surfactants, CDs present advantages, such as improved desorption, non-toxicity, biodegradability and no sorption to the soil particles. For these reasons, the use of CDs have emerged as a useful tool for contaminants removal from soil systems (Villaverde et al., 2018a). Biocompounds produced by fermentative processes appeared as an economic and sustainable alternative to many synthetic molecules (Lopes et al., 2019). Thereby, biosurfactants have become a promising substitute due to their synthesis potential by a wide variety of microorganisms. However, despite their benefits, biosurfactants are not widely used because of the high production costs. Hence, cost-effective substrates, optimized cultivation conditions, and mutant lineage development are imperative to make these biomolecules an economically competitive product to propose a widespread replacement of synthetic surfactants. The data currently available indicate that the cost of production for biosurfactants is between 1 and 60 USD/kg, depending on the degree of purity and product specifications required by the desired application (Morillo and Villaverde, 2017). The feasibility of CDs use concerning the price of bulk material is frequently argued for not selecting them as remediation technology. Gruiz et al. (2009) compared the use of cyclodextrin (RAMEB) as bioavalability enhancer for *in situ* bioremediation with various realistic alternative technologies [monitored natural attenuation, excavation and disposal on landfills, on-site bioventing, soil flushing with water (pump-and-treat)]. They demonstrated that the cost-efficiency was similar or even lower than in the others technologies, proving their efficiency and competitiveness. The estimated cost of this bioremediation technology using CDs was $220/ton, and the duration of the treatment was only 1.5 years (for monitored natural attenuation the cost was $218/ton, but the duration was 15 years). One of the strengths is that CDs technologies are less time consuming than other alternative ones (40–70% lower than the other treatments), and it compensates the price of the bulk material.

The present work studied the isolation and characterization of PAHs degrading bacterial strains from a real contaminated soil (industrial site) with the objective of testing their capacity to be used as a soil bioremediation tool. Therefore, the objective of this study was to prove an effective bioremediation tool based on the inoculation of potential PAHs bacterial degraders previously isolated from soil, coupled with the use of hydroxypropil-β-cyclodextrin (HPBCD), to increase the PAHs bioavailability in order to achieve an improved bioremediation after PYR has been aged in soil.

## Materials and Methods

### Materials

Powdered PAHs (fluorene, FLU, phenanthrene, PHE and pyrene, PYR) were purchased from Sigma-Aldrich (Madrid, Spain). HPBCD was supplied by Cyclolab (Budapest, Hungary). A PAHs contaminated soil from an industrial site was used to obtain potential PAHs microbial degraders (Sánchez-Trujillo et al., 2013). Radiolabeled [ring-^14^C]-PYR was purchased from the Institute of Isotopes, Budapest, Hungary (specific activity 36 mCi mmol^–1^, chemical purity 99.9% and radiochemical purity 100%). There were three different soils (LT, CR, and TM) employed to carry out the biodegradation experiments. They were taken from the superficial horizon (0–20 cm). The soil samples were air-dried for 24 h, stones and plant materials removed, sieved through 2 mm and stored at 20°C. The soils were analyzed for particle size distribution, organic matter, pH and total carbonate content (Table 1).

**TABLE 1:** Some characteristics of the soils used.

**Soils**	**pH**	**CO_3_^–2^ (%)**	**OM (%)**	**Sand (%)**	**Silt (%)**	**Clay (%)**	**Textural classification**
CR	8.0	6.9	0.8	73.9	16.1	10.0	Sandy loam
LT	8.2	21.8	1.3	28.5	45.8	25.7	Loam
TM	8.0	24.1	1.8	2.70	31.5	65.9	Clay
							

### Methods

#### Isolation and Identification of Bacteria From the Contaminated Soil by 16SrRNA Sequence Analysis

The strains employed in this study were isolated from an industrial contaminated soil, which was sampled from an area located at Llaneras (Oviedo, Northern Spain) previously described by Sánchez-Trujillo et al. (2013). The isolates were obtained from both the original contaminated soil and from PHE enriched cultures. The soil (0.3 g) was mixed with 15 mL of sterilized water, and the mixture was shaken for 1 h; after that, 100 μL of the mixture were added in Petri dishes with two different culture media, Tryptone Soya Agar (TSA) and mineral salt basal agar (MSB), and incubated at 28°C for 48 h. Morphologically different bacterial colonies were isolated following standard microbiological protocols and stored in Microbank^TM^ cryovials (2 mL microtubes containing 20 porous spheres of 3 mm diameter) and kept at −80°C (Villaverde et al., 2018b).

PHE enriched cultures were performed with 0.3 g of soil in 15 mL of sterilized water supplemented with PHE (1000 mg L^–1^) and incubated at 30°C for 30 days. Every 2 weeks, 100 μL of the culture was transferred to another flask containing MSB medium plus PHE and incubated again. At the end of the enrichment procedure, 100 μL of this suspension were inoculated in Petri dishes with MSB and incubated at 28°C for 48 h. There were 28 morphologically different bacterial colonies isolated following standard microbiological protocols, stored in Microbank cryovials and kept at −80°C.

DNA from all the isolated bacteria was extracted using an Extraction Kit (Jet quick, PCR TE, Genomed). The 16S ribosomal RNA gene (16S rRNA) was used for bacteria identification. The 16S rRNA gene was amplified by PCR using the primers 616F (5′−AGA GTT TGA TCC TGG CTC AG) and 1510R (5′−GGC TAC CTT GTT ACG ACT T). There were PCR reactions performed in 50 μL volumes, containing 1–2 μL of template DNA, 5 μL of 10 × PCR buffer Biotaq (Bioline, United States), 1.5 μL of 50 mM MgCl_2_ (Bioline), 1 μL of 10 mM deoxyribonucleoside triphosphate mixture (dNTPs) (Invitrogen, Carlsbad, CA, United States), 0.5 μL of 50 μM of each primer and 0.25 μL of *Taq* DNA polymerase enzyme (Bioline, United States), made up to 50 μL with nuclease−free water (Sigma–Aldrich, United States). PCR thermal conditions were as follow: 94°C for 120 s; 35 cycles of 94°C for 20 s, 55°C for 45 s, 72°C for 120 s and a final extension cycle at 72°C for 10 min. The amplified DNA fragment was purified using a purification kit Jetquick PCR Spin kit (Genomed, Löhne, Germany).

Taxonomic identification was done by comparing to NCBI database (National Centre for Biotechnology Information) using the BLASTN algorithm. The sequences were deposited in the NCBI GenBank database with accession numbers JF815694, GQ423064, JF262928 – JF262935, JQ895558, and JQ895559 (Table 2).

**TABLE 2:** Phylogenetic affiliations of bacteria isolated from the contaminated soil.

**Strain (accession number)**	**N° of isolates**	**NCBI affiliation (accession number)**	**Similarity (%)**	**Phylum/Class, family, genus**
JRO (JF815694)^a^	1 (28)	*Advenella kashmirensis* strain WT001 (NR 042360)	99	Betaproteobacteria Alcaligenaceae, Advenella
JR7 (GQ423064)^a^	1 (28)	*Achromobacter xylosoxidans* strain GD003A (MK128503)	99	Betaproteobacteria Alcaligenaceae, Achromobacter
JR52 (JF262928)^a^	16 (28)	*Pseudomonas* sp. strain SA501 (MK294319)	99	Gammaproteobacteria Pseudomonadaceae, Pseudomonas
JR62 (JF262929)^a^	2 (28)	*Stenotrophomonas maltophilia* strain QT24 (GU385870)	99	Gammaproteobacteria Xanthomonadaceae, Stenotrophomonas
JR66 (JF262930)^a^	1 (28)	*Achromobacter spanius* strain UQ283 chromosome, complete genome (CP034689)	99	Betaproteobacteria Alcaligenaceae, Achromobacter
JR73 (JF262931)	1 (28)	*Olivibacter soli* strain Gsoil 034 (NR 041503)	99	Bacteroidetes Sphingobacteriaceae, Olivibacter
5B11 (JF262932)^b^	1 (28)	*Microbacterium* sp. Iso-44 (KC768755)	99	Actinobacteria Microbacteriaceae, Microbacterium
6C32 (JF262933)^a,b^	1 (28)	*Microbacterium oxydans* strain VIU2A chromosome, complete genome (CP031338)	99	Actinobacteria Microbacteriaceae, Microbacterium
6C41 (JF262934)^a,b^	1 (28)	*Acinetobacter lwoffii* strain kp10 (MH200627)	99	Gammaproteobacteria Moraxellaceae, Acinetobacter
6C42 (JF262935)^b^	1 (28)	*Microbacterium oxydans* strain HG3 chromosome, complete genome (CP031422)	99	Actinobacteria Microbacteriaceae, Microbacterium
2BC8 (JQ895559)^a,b^	1 (28)	*Achromobacter xylosoxidans* strain FDAARGOS 150, complete genome (CP014028)	99	Betaproteobacteria Alcaligenaceae, Achromobacter
2BC9 (JQ895558)^a,b^	1 (28)	*Cellulomonas* sp. strain LA6P21 (MG860171)	99	Actinobacteria Cellulomonadaceae, Cellulomonas

*^*a*^Bacteria selected to carry out FLU and PYR biodegradation; ^*b*^Bacteria isolated from PHE enrichment cultures.*

#### Characterisation of Phenanthrene Enriched Cultures by Molecular Analysis

In order to obtain information of phenanthrene enriched cultures, a 16S rRNA gene library was constructed with the TOPO TA Cloning kit (Invitrogen), as described in Gonzalez-Pimentel et al. (2018). The sequences were deposited in the NCBI GenBank database with accession numbers JF262936–JF262945 and JF262952, indicating that 82% of the 16SrRNA sequences analyzed belonged to the class Betaproteobacteria (Table 3). Similar results were observed by Martin et al. (2012) when the microbial community from a soil from a nearby road, which was spiked with PHE, was studied.

**TABLE 3:** Phylogenetic affiliations of the 16S rRNA sequences obtained from PHE enrichment cultures of the contaminated soil.

**Representative clon (accession number)**	**Similar sequences**	**NCBI affiliation (accession number)**	**Similarity (%)**	**Phylum/Class**
E2BCU-KB1 (JF262952)	1	*Alcaligenes* sp. VKM B-2263 (AF430122) 1	98	Betaproteobacteria
E2BCU-KB2 (JF262936)	2	*Achromobacter xylosoxidans* strain APBSMLB83 (MG705862)	99	Betaproteobacteria
E2BCU-KB5 (JF262937)	4	*Advenella* sp. JCM 28249 (LC133596)	98	Betaproteobacteria
E2BCU-KB6 (JF262938)	1	*Achromobacter xylosoxidans* strain APBSMLB83 (MG705862)	99	Betaproteobacteria
E2BCU-KB9 (JF262939)	6	*Advenella kashmirensis* strain 6B1 (MH379789)	99	Betaproteobacteria
E2BCU-KB10 (JF262940)	1	*Achromobacter xylosoxidans* strain FC2996 (MK089550)	99	Betaproteobacteria
E5CCU-KC1 (JF262943)	2	*Advenella kashmirensis* strain 6B1 (MH379789)	99	Betaproteobacteria
E5CCU-KC3 (JF262944)	3	*Advenella kashmirensis* strain 6B1 (MH379789)	99	Betaproteobacteria
E4DCU-KD2 (JF262941)	2	*Achromobacter xylosoxidans* strain FC2996 (MK089550)	99	Betaproteobacteria
E4DCU-KD7 (JF262942)	5	*Pseudomonas alcaliphila* strain CI12 (AB862144)	99	Gammaproteobacteria
E6CCU-KE1 (JF262945)	1	*Advenella* sp. JCM 28249 (LC133596)	99	Betaproteobacteria

#### Isolation and Identification of Bacteria From the Contaminated Soil by 16SrRNA Sequence Analysis

Phylogenetic trees based on the 16SrRNA genes were constructed using the maximum-likelihood (Felsenstein, 1981), neighbor-joining (Saitou and Nei, 1987) and maximum-parsimony algorithms in MEGA7 (Kumar et al., 2016). The tree robustness was evaluated using a boot-strap analysis of 1,000 resamplings (Felsenstein, 1985).

#### Inoculum Preparation

There were six bacterial strains (Table 2) chosen to carry out the biodegradation of PYR and FLU, which were cultivated and subsequently stored in cryovials (Microbank). Cryovials were thawed and the selected bacteria were grown in Luria-Bertani (LB) medium. The bacteria were harvested just after starting stationary phase and washed twice in a sterile MSM solution. The final density of each strain added was 10^8^ CFU mL^–1^.

#### Biodegradation Experiments in Solution

Both FLU and PYR biodegradation in solution, three replicates, were performed in 20 mL glass vials flasks (autoclave Auster-G, P-Selecta with one cycle at 120°C, inlet pressure of 103 kPa, for 20 min) with 15 mL of MSB (g L^–1^): Na_2_HPO.H_2_O (8 g); KH_2_PO_4_ (3 g); NH_4_Cl (1 g); NaCl (0.5 g); MgSO_4_ 1M (1 mL), spiked with 4 mg L^–1^ of PYR or FLU as the only source of C and energy. Vials were inoculated when was required with 300 μL of bacterial culture (10^8^ CFU mL^–1^). Glass vials were kept at a temperature of 20°C in a laboratory oven for 37 days. Throughout this period samples were taken at different times to observe the degradation of the PAHs.

#### Mineralization Assays in Solution and in Soil Suspensions

Mineralization of ^14^C-labeled PYR in solution and in suspension of three soils of different characteristics (both in triplicate) was measured through the evolution of ^14^CO_2_ produced (Villaverde et al., 2012). Mineralization assays were performed in respirometers. The mineralization assays in solution were carried out in modified 250 mL Erlenmeyers as respirometers with 100 mL of MSB, containing ^14^C-ring-labeled and unlabeled PYR to obtain a final concentration of 10 mg L^–1^ in solution. In soil suspension assays, 1 mL of a 1,000 mg L^–1^ PYR stock solution in methanol, which also contained ^14^C labeled PYR, was added to 2.5 g of soil (25% of the total soil used). The solvent was allowed to evaporate for 16 h. The remaining 75% soil was added and mixed to avoid damage to indigenous soil microorganisms, obtaining a final concentration of 100 mg kg^–1^ and a radioactivity of approximately 900 Bq per flask. After that, 100 mL of MSB was added (Reid et al., 1998), and 1 mL of a micronutrient solution (NS) containing trace elements (CaSO_4_⋅2H_2_O, ZnSO_4_⋅7H_2_O, Al_2_(SO_4_)_3_⋅16H_2_O, NiCl_2_⋅ 6H_2_O, CoCl_2_ ⋅ 2H_2_O, KBr, KCl, MnCl_2_⋅ 4H_2_O, SnCl_2_⋅2H_2_O, and FeSO_4_⋅7H_2_O) was also added in both mineralization experiments (Fenlon et al., 2011). Parallel experiments were carried out, inoculating when required with 1 mL of a suspension of *A. xylosoxidans* 2BC8 containing 10^8^ CFU mL^–1^. The flasks were closed with Teflon-lined stoppers and incubated at 20 ± 1°C under shaking. Production of ^14^CO_2_ was measured as radioactivity appearing in the alkali trap of the biometer flasks, which contained 1 mL of 0.5M NaOH. Periodically, the solution was removed from the trap and replaced with fresh alkali. The NaOH solution was mixed with 5 mL of a liquid scintillation cocktail (Ready safe from PerkinElmer, Inc., United States), and the mixture was kept in darkness for about 24 h for dissipation of chemiluminescence. Radioactivity was measured with a liquid scintillation counter (Beckman Instruments Inc., Fullerton, California, model LS5000TD).

The same mineralization experiments were carried out but using only the soil TM which was incubated for 80 days after spiking with unlabeled and ^14^C labeled PYR. No biostimulation or bioaugmentation were added to the soil during this time. Before carrying out mineralization experiments, PYR which could be degraded in soil during this period was measured. An exhaustive extraction of PYR was carried out in the soil samples before and after aging, but less than 5% was lost. After this period, 100 mL of MSB, 1 mL of NS solution containing trace elements and inoculation with AX 2BC8 when required were added and the mineralization procedure was the same as previously described. The percentages PYR degraded along the mineralization were referred to its amount in soil at the beginning of this process.

Mineralization experiments in soil suspension of TM soil aged with PYR were also performed in the presence of HPBCD (20 times the PYR added to soil), with the aim of increasing its bioavailability. The flasks were inoculated, when required, with the specific bacterium *A. xylosoxidans* 2BC8 (1 mL with an initial inoculum density of 10^8^ CFU mL^–1^) (Villaverde et al., 2013) and incubated at 20 ± 1°C.

#### Model of Mineralization Kinetics

Mineralization data were fitted to a first-order equation of the following form (Guerin and Boyd, 1992):

*C*_*t*_ = *C*_0_ e^–^*^*kt*^*, and DT50 = ln 2/*k*

A non-linear regression analysis (Sigmaplot v. 8.0) was used to estimate the kinetic parameter DT50.

#### Enumeration of Bacterial Strain Degraders in Soil

Enumeration of viable bacteria potentially PYR degraders for each soil (CR, LT, and TM) were performed (three replicates). In a 250 mL flask, 10 g of soil were spiked until reaching a PYR concentration of 10 mg kg^–1^, then 100 mL of TSB 0.05× medium was added. The systems were closed and shaken at 23°C for 30 h. There was 100 μL of the solution applied on agar plates prepared from a TSB 0.05× medium, and CFUs were counted after 48 h.

#### Statistical Analysis

Following blank-correction, statistical analysis of the results was performed in SigmaStat for windows (Version 2.03, SPSS Inc.). Significant effects on the mineralization of 14C-PYR in soil slurry (three different studied soils) and in inoculated and non-inoculated solution were compared statistically using a General Linear Model (ANOVA) (three replicates) (Tukey test, *P* < 0.05).

## Results and Discussion

### Isolation and Characterization of Potential PAHs Degraders Present in the Contaminated Soil

There were 28 strains isolated from the original contaminated soil and from PHE enrichment cultures (Table 2). These strains were differentiated by their morphology, as indicated in previous section. 16S rRNA gene sequences of all twenty-eight bacteria showed a match of 99% to those from bacteria in the NCBI GenBank database. There were 19 strains (68%) belonging to the class *Gammaproteobacteria* (*Pseudomonas* sp., *Stenotrophomonas* sp., and *Acinetobacter* sp.), four strains belonging to the class *Betaproteobacteria* (*Ahromobacter* sp., *Advenella* sp.), another four strains belonging to the phylum *Actinobacteria* (*Microbacterium* sp., *Cellulomonas* sp.) and only one strain belonging to the Bacteroidetes (*Olivibacter* sp.), concluding that 23 isolates belong to the phylum *Proteobacteria* (Delgado-Baquerizo et al., 2018). This result is in accordance with those observed by Labbé et al. (2007) and Cebrón et al. (2009) in hydrocarbon contaminated soils, which observed that the majority of microorganisms belonged to *Gammaproteobacteria* and *Betaproteobacteria*.

**FIGURE 1:**
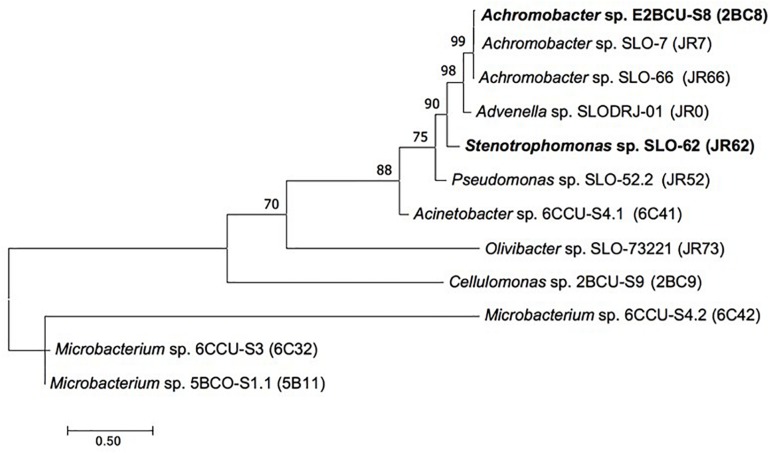
Maximum Likelihood tree based on 16S rRNA gene showing the relationships between the strains *Achromobacter xylosoxidans* 2BC8 and *Stenotrophomonas maltophilia* JR62 and other isolated bacteria obtained from the original soil or from the enrichment culture. Bootstrap values (>70%) are expressed as percentages of 1000 replicates. Same branches representation was recovered by the neighbour-joining and maximum-parsimony algorithms. The scale bar indicates 0.50 substitutions per nucleotide position in 16S rRNA gene sequences tree.

Figure 1 shows Maximum Likelihood tree based on 16S rRNA gene showing the relationships between the FLU and PYR degrading strains (2BC8 and JR62) and other isolated bacteria from the enrichment culture.

The studied soil showed a high level of contamination of Σ16 PAHs 1068 ± 101 mg kg^–1^ (Sánchez-Trujillo et al., 2013). This soil has been contaminated for more than 20 years, and hence, these strains have been exposed to this contamination over a long time, which indicates that these microorganisms have been capable of adapting their metabolism in the presence of the PAHs, using them as carbon source (Serrano et al., 2009). Haleyur et al. (2018) identified different bacterial strains previously isolated from aged weathered soils in an Australian former gasworks site, showing the catabolic potential of indigenous bacteria for PAHs-degradation.

*Pseudomonas* has been the most frequently found in the studied strains. The identified genus has been previously described as a potential PAHs degrader in literature, isolated from sites affected by significant levels of contamination by hydrocarbons (Haritash and Kaushik, 2009; Ghosal et al., 2016). Zhao et al. (2009) determined that there are a wide variety of bacterial strains capable of degrading PAHs, in particular low weight PAHs, isolated from contaminated soils. Among the identified, *Acinetobacter* sp., *Pseudomonas* sp., and *Stenotrophomonas* sp. prevailed. The genus *Acinetobacter* sp. has been reported in literature as a PAHs degrader (Yu et al., 2005; Janbandhu and Fulekar, 2011; Kafilzadeh et al., 2011). Kostka et al. (2011) isolated 24 bacterial strains belonging to 14 different genera, including Acinetobacter, from beach sand affected by oil discharge.

Zhao et al. (2011) tested the capacity to degrade hydrocarbons of a bacterial consortium obtained from a contaminated soil in an oil reservoir, where *Microbacterium* sp. was present. Schippers et al. (2005) isolated three oil degrading bacterial strains, which were identified as three new strains of the genus *Microbacterium* sp.

The role of different strains belonging to the genus *Cellulomonas* sp. in bioremediation of polluted soils by hydrocarbons has been also reported. Al-Awadhi et al. (2007) identified different bacterial strains of *Cellulomonas* sp. capable of degrading both aliphatic and aromatic hydrocarbons. Brito et al. (2006) carried out a microbial characterization of the PAHs degrading bacterial communities isolated from the mangrove sediments and they identified a *Cellulomonas* sp. as PYR degrader.

The genus *Olivibacter* sp. was identified and characterized for first time during the process of production of olive oil (Ntougias et al., 2007). Different species of the genus *Olivibacter* can be found in contaminated soils, wastes and cave environments, which can be able to degrade complex and toxic chemicals (Ntougias et al., 2015). Szabó et al. (2011) isolated a novel strain capable of degrading hydrocarbons, *Olivibacter oleidegradans*, from a clean-up facility (biofilter) in a hydrocarbon contaminated site.

The ability of *Stenotrophomonas* sp. to degrade PAHs and other hydrocarbons has been demonstrated (Tiwari et al., 2016; Nowak and Mrozik, 2018) and in particular, *S. maltophilia* strains (Chowdhury et al., 2017; Kumari et al., 2018). The strain *S. maltophilia* AJH1 was able to degrade low and high molecular weight PAHs (up to 95 and 80%, respectively) in acidophilic medium at pH 2 (Arulazhagan et al., 2017). Zafra et al. (2014) selected a degrading microbial consortium isolated from crude oil contaminated soils (50 fungal and bacteria isolates), highly tolerant to three-, four-, and five-ring PAHs, and *S. maltophilia* B14 grew employing PAHs as the sole carbon source and presented a high tolerance to PAHs up to 6 g L^–1^. Juhasz et al. (2000) proved the ability of *S. maltophilia* strain VUN 10,003 to degrade PYR, FLU, benz[a] anthracene, benzol[a] pyrene, dibenz[a,h]anthracene and coronene.

In relation to *Achromobacter* sp., Janbandhu and Fulekar (2011) reported, for the first time, that the novel bacterium *Achromobacter insolitus* MHF ENV IV degraded PHE; Ma et al. (2015) observed the degradation of fluoranthene by *A. xylosoxidans* DN002 isolated from a petroleum-contaminated soil. Dave et al. (2014) observed an enhanced biodegradation of total polycyclic aromatic hydrocarbons (TPAHs) when using *A. xylosoxidans*, isolated from crude oil polluted marine sites. Gunasekera et al. (2018) isolated a *Achromobacter spanius* strain which was present in the hydrocarbon-degrading bacterial community in a desert soil sample obtained under a fuel bladder.

### FLU and PYR Biodegradation in Solution

A gen bank was created with the genes that codified by 16S RNA amplified by polymerase chain reaction (PCR) of the isolated 28 strains (Table 2). These strains were cultivated, although it was not possible to obtain enough microbial biomass of *Olivibacter soli* JR73 to be used in biodegradation processes. The most representative potential PYR degraders strains found in the studied soil were selected to perform FLU and PYR biodegradation assays in solution: *Advenella* sp. JRO and *Achromobacter* sp. 2BC8 (Betaproteobacteria); *Stenotrophomonas* sp. JR62 (SM) and *Acinetobacter* sp. 6C41 (Gammaproteobacteria) and *Microbacterium* sp. 6C32 and *Cellulomonas* sp. 2BC9 (Actinobacteria). *Pseudomonas* spp. (Betaproteobacteria) was not selected since it is a very known PAH degrader (Sopeña et al., 2013). Only two of the six strains selected showed a significant biodegradation capacity for FLU and PYR (Supplementary Figure S1). With *A. xylosoxidans* 2BC8 inoculum, FLU reached a biodegradation of about 45%, while when *S. maltophilia* JR62 was inoculated, a complete biodegradation of FLU was observed. The percentage of PYR biodegraded by *A. xylosoxidans* 2BC8 reached about 40% during the experimental period, while with *S. maltophilia* JR62, the PYR biodegraded was 32%.

The selected strains were identified as *A. xylosoxidans* 2BC8 and *S. maltophilia* JR62, both showing an identity of 99% with accession numbers CP014028.2 and GU385870.1, respectively. Other authors have also reported PAHs degradation using *A. xylosoxidans* and *S. maltophilia*, as it has been mentioned before, but they are quite uncommon strains for PAHs degradation. Castro-Gutiérrez et al. (2012) isolated sixteen PAH-degrading strains with the ability to grow on naphthalene (NAP), PHE, FLU, and PYR. Most of the isolates belonged to the genus *Pseudomonas*, although *Comamonas*, *Sphingomonas*, Stenotrophomonas and *Delftia* were also found. Tiwari et al. (2010) studied a bacterium characterized as *A. xylosoxidans*, isolated from an oil refinery effluent sludge and capable of aerobic degradation of PYR. Ghevariya et al. (2011) isolated a multiple PAH degrading halotolerant *A. xylosoxidans*, which was isolated from crude oil polluted saline site and exhibited 86% chrysene degradation. As far as authors know, the scarce studies carried out using *A. xylosoxidans* as a degrader of PAHs have arisen after 2010.

### PYR Mineralization in Solution

**FIGURE 2:**
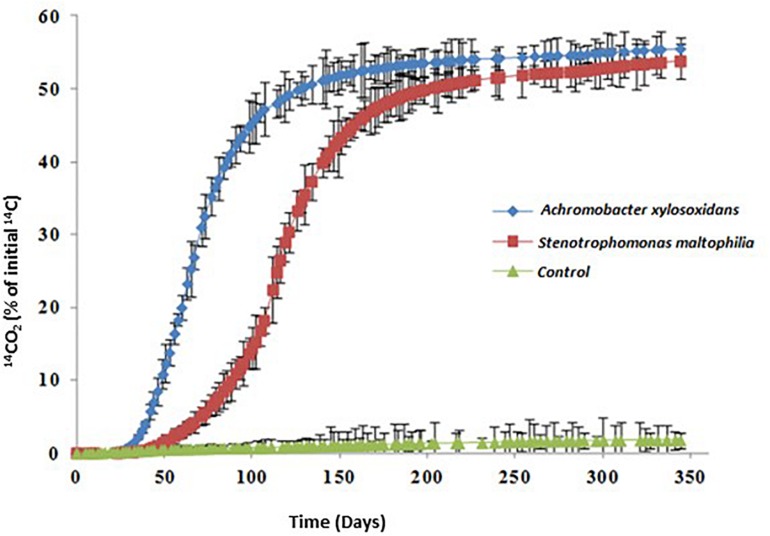
PYR mineralization curves in solution in the presence of *Achromobacter xylosoxidans* 2BC8 and *Stenotrophomonas maltophilia* JR62.

PYR is considered one of the priority organic contaminants due to its toxicity and persistence. One of the strategies followed to improve organic pollutant biodegradation consists of using isolated microbial consortia due to their capacity for synergistic metabolism, avoiding potential toxic effects of the metabolites formed. However, the use of a single mineralizing microorganism, from a scientific point of view, is more interesting in order to control the different factors that influence in the effectiveness of bioaugmentation treatment and to determine the pollutant biodegradation metabolic pathway. For these reasons, isolation of a single degrader strain can be considered a first step to optimize an effective bioaugmentation tool for PAHs bioremediation. The two strains isolated and identified in this work (*A. xylosoxidans* 2BC8 and *S. maltophilia* JR62) were also proved for PYR mineralization. Figure 2 shows PYR mineralization curves obtained after their inoculation. Authors have concluded that inoculum density at a level of 10^6^–10^10^ UFC g^–1^ of soil is suitable for the efficient degradation of organic contaminants by the microorganisms inoculated (Alexander, 2000). However, some authors observed an initial lag-phase in an organic pollutant biodegradation profile after inoculation of microorganisms at an even higher level (Cycoń et al., 2014).

The tested strains were capable of mineralizing PYR (Table 4). *A. xylosoxidans* 2BC8 was able to adapt to use PYR as carbon source quicker than *S. maltophilia* JR62 (40 and 69 days of acclimation period, respectively). Analysis of variance when the only factor study was soil indicated that there were only significant differences in the acclimation period and DT50.

**TABLE 4:** Parameters obtained from PYR mineralization in solution after inoculation with *Achromobacter xylosoxidans* 2BC8 and *Stenotrophomonas maltophilia* JR62.

	**Acclimation period (days)**	**Extent of mineralization (%)**	**Mineralization rate (% day^–1^)**	**DT50 (days)**
AX 2BC8	40.0 (3.5)	55.5 (1.5)	1.00 (0.22)	118 (4)
SM JR62	69.1 (4.5)	53.8 (2.4)	1.08 (0.18)	200 (8)
ANOVA (*p*)^∗^	0.001	0.361	0.651	0.000

*Standard deviation in parenthesis (*n* = 3). ^∗^ANOVA GLM – Homogeneity of variance by Levene test (*p* > 0.05), ANOVA test LSD.*

The mineralization rate was similar for both strains, 1.00 and 1.08% days^–1^. Significant differences between the values reached for the PYR extent of mineralization were not found (55.5 and 53.8%, respectively). By contrast, *A. xylosoxidans* was able to mineralize 50% of PYR in solution faster than *S. maltophilia* JR62 (DT50 118 and 200 days, respectively).

Numerous microorganisms capable of degrading low and medium weight PAHs have been isolated. It is important that these microorganisms do not form toxic metabolites during the PAH degradation, and for this reason, it is important to determine their pollutant mineralization capacity (Guo et al., 2017; Isaac et al., 2017; Aziz et al., 2018), and therefore, soil toxicity evolution during the biodegradation process should be also analyzed (Molina et al., 2009).

From the two selected strains, *A. xylosoxidans* 2BC8 was chosen for the subsequent soil mineralization studies due to its higher capacity to adapt to the presence of PYR in the medium (lower acclimation period) and DT50, and also because there are very few published works which have demonstrated the effectivity of *Achromobacter* sp. as PAH degrader, as commented previously.

### PYR Mineralization in Contaminated Soils

**FIGURE 3:**
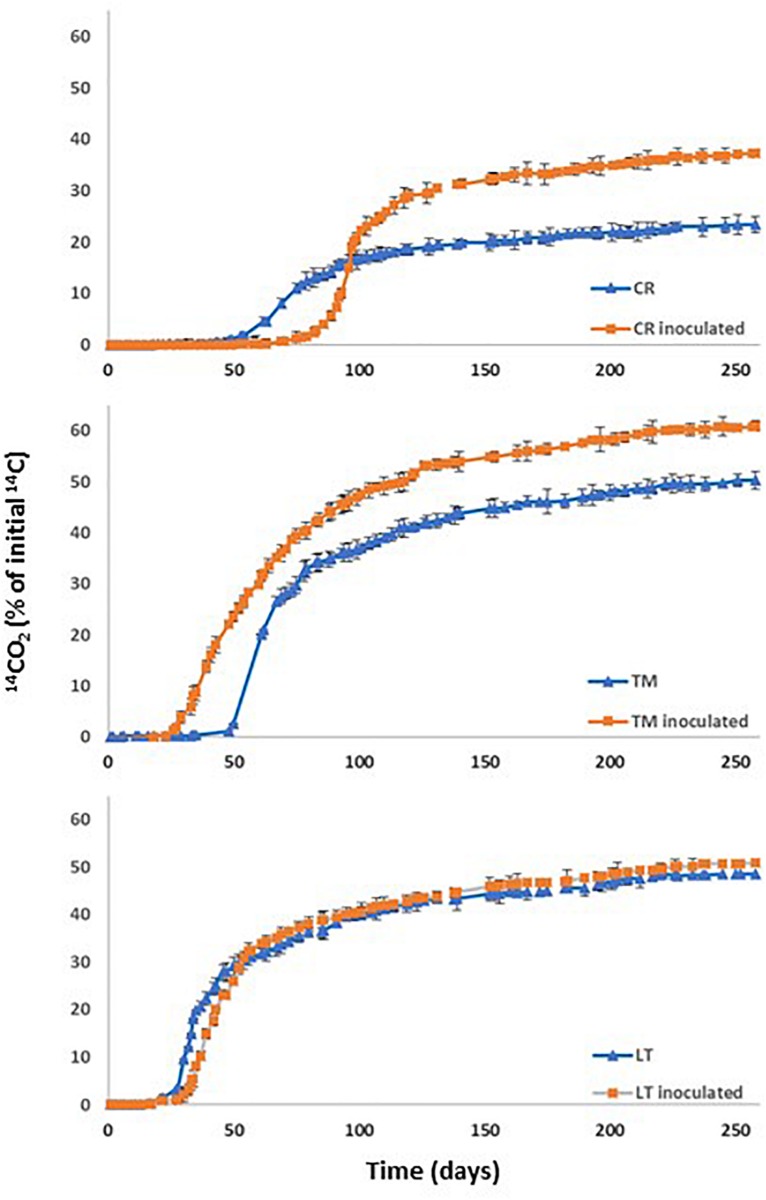
PYR mineralization curves in non-sterilised soil CR, LT, TM without inoculation and after inoculation with *Achromobacter xylosoxidans* 2BC8.

With the aim of determining the natural attenuation capacity of soils non-exposed previously to PAHs contamination, three soils with different properties (Table 1) were selected and spiked with PYR (10 mg kg^–1^). Soil mineralization curves corresponding to the soils named as CR, LT, and TM are shown in Figure 3. For all the studied soils significant differences in acclimation period of the endogenous microbiota were observed between them (Table 5). When the factors soil and inoculation were separately taken into account, the statistical analysis indicated that they have effect over all the studied variables. Interaction of both factors (soil and inoculation) indicated that the effect of the inoculum depended of the type of soil (Supplementary Table S1). However, although the extent of mineralization was similar for the soils LT and TM, the PYR mineralization rate was the highest in the case of soil LT. Regarding DT50 values obtained in Table 5, this value was not reached for CR and LT soils during the assay time and in the case of TM soil the calculated value was 258 days.

**TABLE 5:** Parameters obtained from PYR mineralization in soils before and after their inoculation with *Achromobacter xylosoxidans* 2BC8 (AX 2BC8).

	**Acclimation period (days)**	**Extent of mineralization (%)**	**Mineralization rate (% day^–1^)**	**DT50 (days)**
CR	62.0 (5.7)c	23.3 (1.5)a	0.56 (0.07)a	–
LT	28.2 (3.8)a	48.8 (1.8)c	2.47 (0.11)d	–
TM	49.6 (3.5)b	50.0 (2.5)c	1.43 (0.10)b	258 (9)
CR inoculated	90.0 (5.4)d	36.7 (1.8)b	2.16 (0.20)cd	–
LT inoculated	34.7 (4.9)a	51.4 (1.4)c	1.87 (0.31)bc	225 (9)
TM inoculated	32.4 (3.3)a	60.5 (1.5)d	1.66 (0.27)bc	120 (7)

*Standard deviation in parenthesis (*n* = 3). ANOVA GLM – Homogeneity of variance by Levene test (*p* > 0.05), ANOVA test LSD [(Two ways, *p* < 0.05) (One way, *p* < 0.05)]; The same lower case letter indicates no statistically significant differences of means (Tukey Test HSD, *p* > 0.05). See Supplementary Table S1.*

Soil natural attenuation assessments determined for the three investigated soils were correlated with the enumerated soil PYR microbial degraders in each soil (CFU g^–1^ of soil). The soils, LT and TM, which showed a similar extent of PYR mineralization, presented 8 × 10^7^ and 2 × 10^7^ CFU g^–1^, respectively, of potential PYR bacterial degraders. CR soil showed the longest acclimation period (62 days) and the lowest extent of mineralization (23.3%) and mineralization rate (0.56% days^–1^), which is clearly correlated with the lowest concentration of soil PYR potential degraders (8 × 10^6^ CFU g^–1^). Towell et al. (2011) investigated the microbial degradation of ^14^C-labeled hexadecane, octacosane, PHE and PYR and observed that soils with the highest concentration of microbial PYR degraders showed the highest mineralization rates. Wu et al. (2016) concluded that the populations of the total petroleum hydrocarbons (TPH) degraders in soil were positively correlated to TPH biodegradation efficiency during bioremediation. Simarro et al. (2013) assessed the effectiveness of different *in situ* bioremediation treatments (bioaugmentation, biostimulation, bioaugmentation and biostimulation, and natural attenuation) on creosote polluted soil, and results showed that creosote decreased significantly in all treatments, and no significant differences were found between treatments. However, some specific polycyclic aromatic hydrocarbons (PAH) were degraded to a greater extent by biostimulation.

Bioaugmentation of the contaminated soil with the strain *A. xylosoxidans* 2BC8 was performed with the aim of showing its potential for PYR biodegradation. PYR mineralization curves after the inoculation of CR, LT and TM soils are also shown in Figure 3 and mineralization results in Table 5. In all cases, the global extent of PYR mineralization was improved with bioaugmentation regarding when the soils were not inoculated. Inoculation in soils CR and TM led to an important increase of the global extent of mineralization, regarding that obtained with the autochthonous soil microbiota (57.5 and 21% of increase, respectively). In LT, soil inoculation had no significant effect in the global extent of mineralization, increasing only about 4.5%. The fact that LT soil had the highest autochthonous PYR bacterial degraders (8 × 10^7^ UFC g^–1^) could result in a higher competition with the *A. xylosoxidans* 2BC8 inoculated, resulting in a similar PYR biodegradation profile. Among the biotic factors affecting bioaugmentation, the most important seem to be the interactions between autochthonous and inoculated microorganisms, such as predation and the competition for nutrients and niches. However, it is thought that the most important factor influencing the success of bioaugmentation is the ability of inoculants to survive in the contaminated environment (Cycon et al., 2017). On the contrary, soil CR had the lowest concentration in specific PYR degraders (8 × 10^6^ g^–1^), which makes the effect of *A. xylosoxidans* 2BC8 inoculation more notorious. Villaverde et al. (2017) observed a competitive effect between the inoculum and soil endogenous microbiota when a diuron degrading microbial consortium was inoculated in six different soils. Cycon et al. (2017) indicated that it has been observed that when lower inoculum densities (<10^4^ CFU g^–1^ of soil) were used, only a small part of the introduced bacteria survived the initial competition and participated in pesticide degradation. In our case, the final density of each strain added was 10^7^ CFU g^–1^ of soil, what would secure an increase in the period of effectiveness of the inoculate. Maddelaa et al. (2016) conducted a study a field-level to determine total petroleum hydrocarbon (TPH)-degrading potential of two bacterial strains, Bacillus thuringiensis B3 and B. cereus B6, and two fungi, Geomyces pannorum HR and Geomyces sp. strain HV, all soil isolates obtained from an oil field located in north-east region of Ecuador. Crude oil-treated soil samples contained in wooden boxes received a mixture of all the four microorganisms and were incubated for 90 days in an open low-land area of Amazon rainforest and the bacteria mixed inoculum density used was 0.1 × 10^6^ cfu g^–1^.

### Aging Effect on Soil PYR Mineralization

**FIGURE 4:**
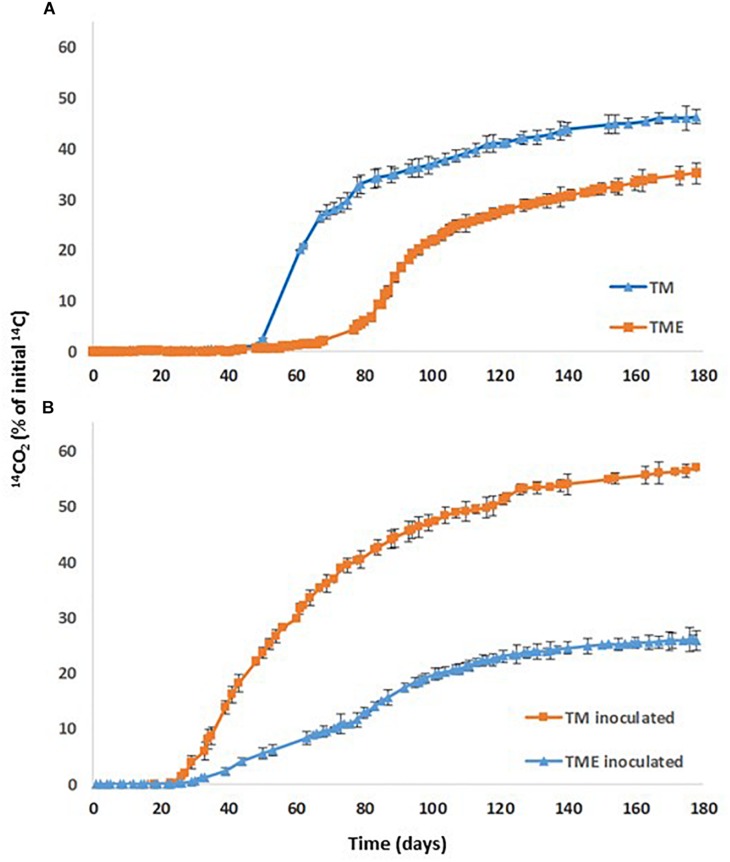
PYR mineralization curves in: **(A)** TM soil (TM) and TM soil after aging (TME); **(B)** TM soil inoculated with *Achromobacter xylosoxidans* 2BC8 (TM inoculated) and TM soil inoculated after aging (TME inoculated).

Soil TM was selected for this study due to its high content in organic matter and clay fraction. It was spiked with PYR (10 mg kg^–1^) and aged for 80 days. In Figure 4A, the aging effect on PYR mineralization in the soil is shown. An increase in the acclimation period from 49 days for TM soil to 79 days for aged TM soil (TME), a decrease in mineralization rate (from 1.43 to 1.20% d^–1^), and the total extension of mineralization (from 46 to 35%) was observed (Table 6). Aging of organic chemicals in soils has been described by several mechanisms involved, including non-desorbed fraction of the chemical molecules within some components of soil organic matter (Brusseau et al., 1994) and organic molecules can diffuse slowly into organic matter soil matrix and be entrapped within small pores (Villaverde et al., 2009; Cheng et al., 2012; Ran et al., 2013). The aging effect is described as the trapping of organic pollutants, reducing their bioaccessibility. It is also possible that strong bonds may form between organic pollutants and soil organic matter (Kong et al., 2013). Rubio-Bellido et al. (2018) studied the aging of diuron contaminated soil for 100 days and observed a drastic decrease of diuron desorbed and mineralized fraction.

**TABLE 6:** Parameters obtained from PYR mineralization in TM soil (TM), TM soil after aging (TME), TM soil inoculated with AX 2BC8 (TM inoculated), TM soil inoculated after aging (TME inoculated), TM soil treated with HPBCD after aging (TME + HPBCD), and TM soil inoculated and treated with HPBCD after aging (TME inoculated + HPBCD).

	**Acclimation period (days)**	**Extent of mineralization (%)**	**Mineralization rate (% day^–1^)**
TM	49.0 (3.5)b	46.1 (3.1)d	1.43 (0.10)cd
TME	79.2 (4.0)c	35.1 (3.1)c	1.20 (0.14)bc
TM inoculated	32.1 (4.9)a	60.5 (1.5)e	1.66 (0.27)d
TME inoculated	45.3 (2.7)b	27.3 (1.7)ab	0.58 (0.06)a
TME + HPBCD	54.4 (2.9)b	33.9 (2.9)bc	1.00 (0.11)b
TME inoculated + HPBCD	46.0 (4.4)b	25.0 (1.5)a	0.86 (0.08)ab

*Standard deviation in parenthesis (*n* = 3). ANOVA GLM – Homogeneity of variance by Levene test (*p* > 0.05), ANOVA test LSD (One way, *p* < 0.05 for the combined effect of the factors Inoculation, Aging and HPBCD); The same lower case letter indicates no statistically significant differences of means (Tukey Test HSD, *p* > 0.05). See Supplementary Table S2.*

**FIGURE 5:**
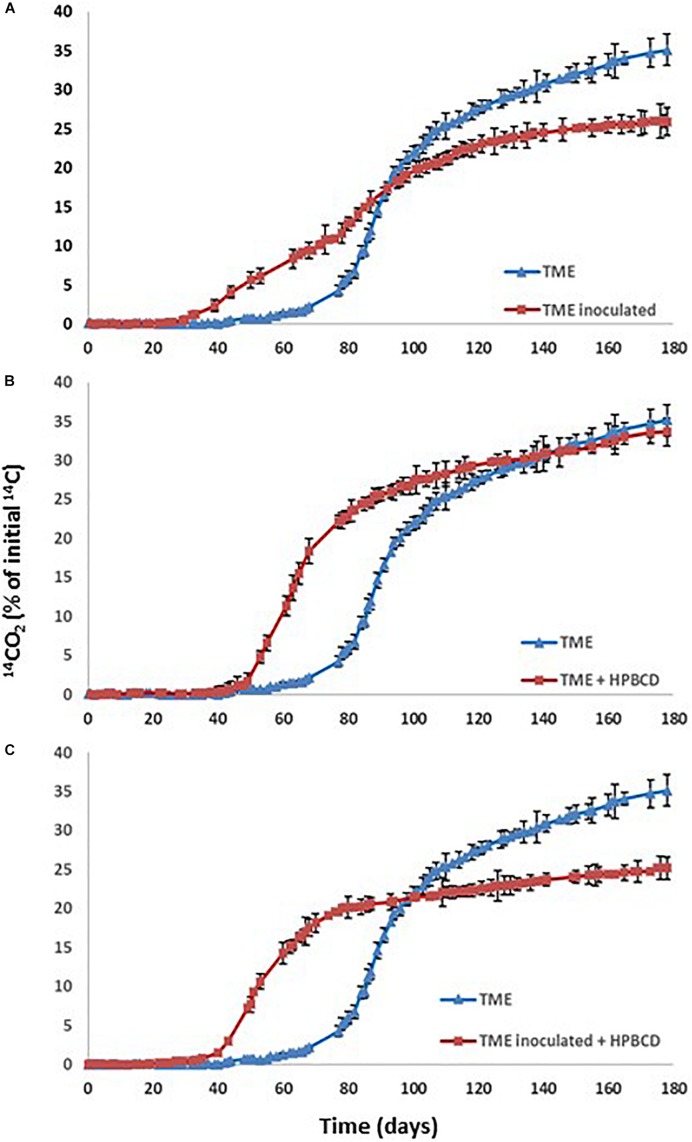
PYR mineralization curves in aged TM soil (TME): **(A)** Effect of the inoculation with *Achromobacter xylosoxidans* 2BC8; **(B)** Effect of HPBCD addition; **(C)** Effect of HPBCD addition combined with *Achromobacter xylosoxidans* 2BC8.

When PYR is aged in the studied soil and after 80 days it is inoculated with *A. xylosoxidans* 2BC8 (Figure 4B), the acclimation period was significantly reduced from 79 to 45 days (Table 6 and Figure 5A), but this was not translated into an enhancement of the total extent of PYR mineralization results, unlike when PYR is not aged in the soil. The presence in the soil TM of specific PYR degraders and the increasing of the contact time between PYR molecules and microbial degraders will contribute to a catabolic induction, producing specific enzymes for PYR degradation and giving rise to a reduced acclimation period due to this previous activation (Fenlon et al., 2011). However, the lower mineralization observed after inoculation with *A. xylosoxidans* 2BC8 of the aging soil TME seems to indicate the strong microbial competence established between the endogenous soil flora capable of degrading PYR, already activated due to the aging, and the inoculated *A. xylosoxidans* 2BC8 strain. A similar competence was observed by Villaverde et al. (2017) in the bioremediation of diuron contaminated soils by a novel degrading microbial consortium.

### Effect of HPBCD Application on PYR Mineralization in Soil

Bioremediation of contaminated soil has been improved with the use of CDs due to their ability to increase the water solubility of hydrophobic organic compounds, facilitating their mobilization (Morillo and Villaverde, 2017). A way of increasing the biodegradable fraction of POPs (persistent organic pollutants) in soils is this movement of the contaminant molecules from the soil into soil solution (Morillo et al., 2014; Sánchez-Trujillo et al., 2014; Madrid et al., 2016, 2019; Simpanen et al., 2016; Guo et al., 2017). CDs have been proposed as an alternative to synthetic surfactants for the removal of hydrophobic contaminants from soils (Fenyvesi et al., 2009). Synthetic surfactants have the disadvantage of being toxic to the resident microbial population. Moreover, surfactants use to form high-viscosity emulsions difficult to remove from soil due to their low water-solubility, and sometimes the adsorption of surfactants onto soils is high. The advantage of using CDs as extractants is their low environmental impact. CDs have been approved as non-toxic compounds that do not harm resident microbial populations. In addition, due to their glucose-based composition, CDs are considered biodegradable, although some CDs are resistant for at least a few months (Fenyvesi et al., 2005).

The tested CD in this work (HPBCD) was proved for use as a carbon source by *A. xylosoxidans* 2BC8 because if this strain was capable of metabolizing HPBCD, it would be highly likely that it would be employed as a carbon source instead of PYR. The studied strain grew in a similar way in the absence (control) and the presence of HPBCD, indicating that the selected strain does not use HPBCD as a carbon source or energy. Therefore, when *A. xylosoxidans* 2BC8 in the PYR mineralization assays in soil is inoculated, the strain will not prefer to biodegrade HPBCD.

In PYR mineralization assays, HPBCD was added to the TM soil after PYR aging for 80 days, with the aim of simulating a more realistic scenario of soil contamination and PYR bioaccessibility evaluation. The PYR mineralization profile in the presence of HPBCD is shown in Figure 5B and data from the different kinetic profiles in Table 6. CDs are able to increase the hydrosolubility of different PAHs, as observed in previous studies through the formation of inclusion complexes (Morillo et al., 2012). HPBCD addition (Figure 5B) provoked a significant decrease of 25 days in the acclimation period (from 79 days for TME to 54 days for TME + HPBCD) (Table 6) in relation to the mineralization in the absence of HPBCD, although a similar global extension of mineralization and mineralization rate was determined (33.9 and 1.00% day^–1^, respectively). The explanation for this reduction is the higher bioaccessibility of PYR after HPBCD application due to an increase of its bioavailable fraction just from the beginning of the assay (Reid et al., 2000), reaching a similar global extension of mineralization corresponding to the global PYR bioavailable fraction in soil (Rubio-Bellido et al., 2016). These results also reveal that the increase of PYR bioavailability due to HPBCD addition has no toxic effects on the indigenous microbiota.

Figure 5C shows the PYR mineralization profiles in the aged soil when *A. xylosoxidans* 2BC8 inoculation and HPBCD were jointly applied. The extent of PYR mineralization was reduced to 25% (Table 6). The statistical analysis indicated that the effect of inoculation depended on the presence or absence of HPBCD (Supplementary Table S2). In this case, besides the competitive effect of endogenous microbiota with *A. xylosoxidans* 2BC8 strain previously mentioned, the application of HPBCD increased PYR bioavailability, and hence its concentration in the soil suspension, what would give rise to a toxic effect on the exogenous inoculated bacterium. Stroud et al. (2009) observed that the amendment of HPBCD to phenanthrene and hexadecane contaminated soil did not enhance biodegradation after 25 days of aging because CD interfered with microbial degradation, which resulted in a lower biodegradation extent.

## Conclusion

From the 28 strains isolated from the original contaminated soil and from the enrichment cultures, two of them, *A. xylosoxidans* 2BC8 and *S. maltophilia* JR62 (uncommon bacterial strains capable of degrading PAHs) demonstrated their capacity to degrade fluorene and pyrene in solution. Both strains were also capable of mineralizing pyrene in solution, but *A. xylosoxidans* 2BC8 was faster and was selected to carry out bioaugmentation of three pyrene contaminated soils. The global extent of mineralization was improved in all cases in the bioaugmented soils, but the increment was less pronounced as the number of autochthonous pyrene bacterial degraders in the soil was greater. Bioaugmentation with *A. xylosoxidans* 2BC8 in aged pyrene contaminated TM soil increased the acclimation period, and decreased the total extent of mineralization, in comparison to the non-inoculated PYR aged soil, indicating a stronger microbial competence between the indigenous soil flora specific degrader of PYR, activated due to the aging, and the inoculated exogenous strain. The same competitive effect between indigenous and exogenous flora was also observed when HPBCD was added as extractant to increase pyrene bioavailability. All these results indicate that the effect of the addition of exogenous bacterial strains for the remediation of contaminated soils will depend on the specific microbiological characteristics of the soil and the positive or negative impact of the new strains on it. And the same occurs with the use of extractants such as HPBCD, since the increase of the contaminant bioavailability could have negative toxic effects on both the indigenous and the exogenous microbiota.

## Data Availability Statement

All datasets generated for this study are included in the article/Supplementary Material.

## Author Contributions

JV, LL, and EM designed research. JV, LL, EM, and AL-M performed research. JV, LL, EM and JG-P analyzed data. JV, LL, EM, JG-P and AL-M wrote the manuscript. All authors read and approved the final manuscript version.

## Conflict of Interest

The authors declare that the research was conducted in the absence of any commercial or financial relationships that could be construed as a potential conflict of interest.
