# Supplementary material for: Bioaugmentation of PAH-Contaminated Soils With Novel Specific Degrader Strains Isolated From a Contaminated Industrial Site. Effect of Hydroxypropyl-β-Cyclodextrin as PAH Bioavailability Enhancer
Source: Front Microbiol. 2019 Nov 14;10:2588. doi: 10.3389/fmicb.2019.02588 (PMC6874150; doi:10.3389/fmicb.2019.02588)
Supplement: Supplementary file 1 [file Image_1.pdf]

FIGURES

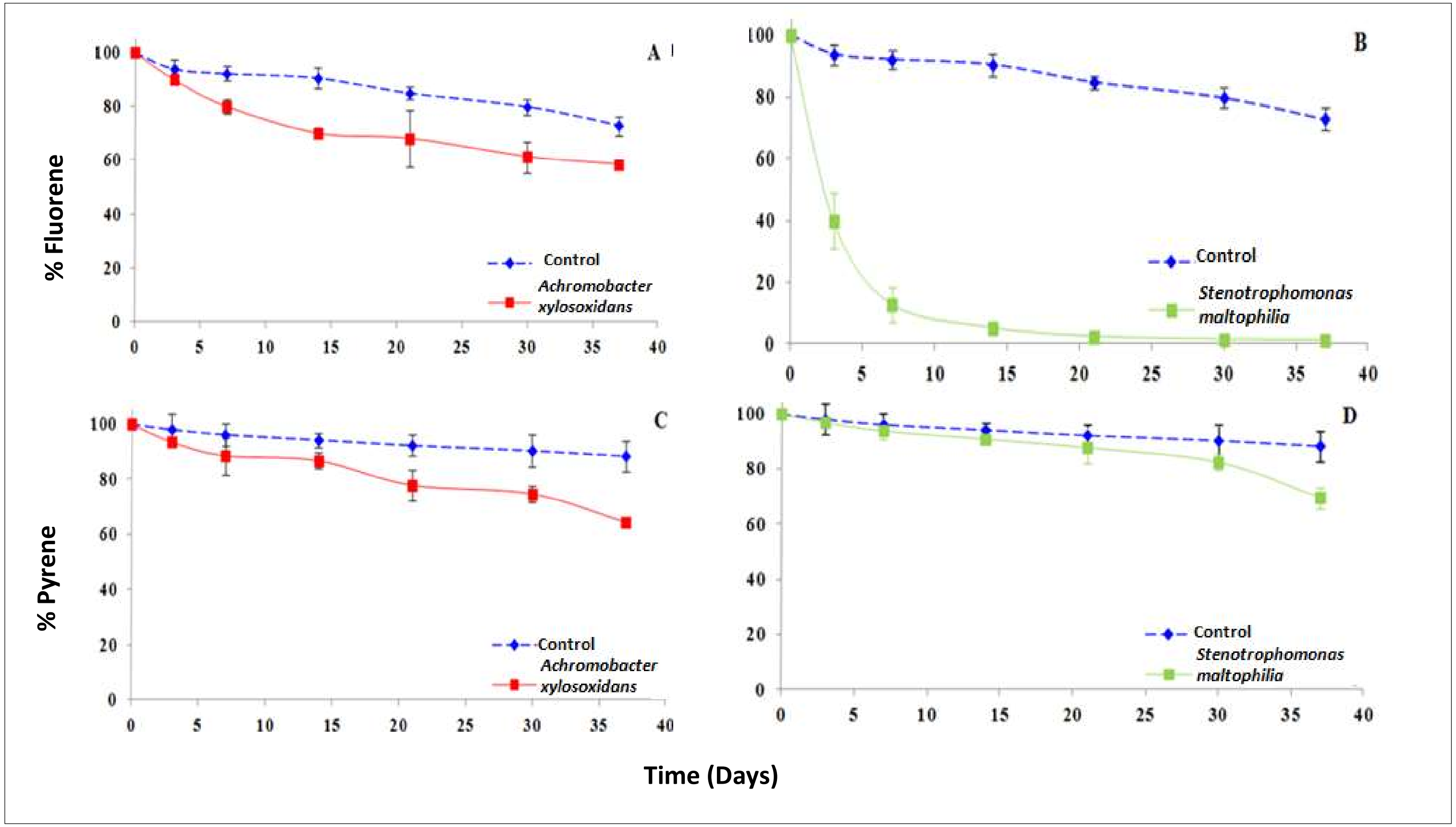

FIGURE S1 PYR and FLU biodegradation curves in solution in the presence of *Achromobacter xylosoxidans* and *Stenotrophomonas maltophilia*.
