# Supplementary material for: Bioaugmentation of PAH-Contaminated Soils With Novel Specific Degrader Strains Isolated From a Contaminated Industrial Site. Effect of Hydroxypropyl-β-Cyclodextrin as PAH Bioavailability Enhancer
Source: Front Microbiol. 2019 Nov 14;10:2588. doi: 10.3389/fmicb.2019.02588 (PMC6874150; doi:10.3389/fmicb.2019.02588)
Supplement: Supplementary file 2 [file Table_1.docx]

|  | **Acclimation period (days)** | **Extent of mineralization (%)** | **Mineralisation rate (% day^-1^)** | **DT50 (days)** |
| --- | --- | --- | --- | --- |
| CR | 62.0 (5.7) c | 23.3 (1.5) a | 0.56 (0.07) a | - |
| LT | 28.2 (3.8) a | 48.8 (1.8) c | 2.47 (0.11) d | - |
| TM | 49.6 (3.5) b | 50.0 (2.5) c | 1.43 (0.10) b | 258 (9) |
| CR inoculated | 90.0 (5.4) d | 36.7 (1.8) b | 2.16 (0.20) cd | - |
| LT inoculated | 34.7 (4.9) a | 51.4 (1.4) c | 1.87 (0.31) bc | 225 (9) |
| TM inoculated | 32.4 (3.3) a | 60.5 (1.5) d | 1.66 (0.27) bc | 120 (7) |
| ANOVA TWO WAYS*  (GLM) (*p*-value) |  |  |  |  |
| Effect of Inoculum (I) | 0,022 | 0,000 | 0,001 | - |
| Effect of Soil (S) | 0,000 | 0,000 | 0,000 |  |
| Interaction (I x S) | 0,000 | 0,001 | 0,000 |  |
| ANOVA one way**  Effect (I x S) | 0,000 | 0,000 | 0,000 |  |

**TABLE S1.** Parameters obtained from PYR mineralisation in soils before and after their inoculation with *Achromobacter xylosoxidans* 2BC8 (AX 2BC8). Standard deviation in parenthesis (n = 3). ANOVA GLM.

* Homogeneity of variance by Levene test (p > 0.05), ANOVA test LSD.

** The same lower case letter indicates no statistically significant differences of means (Tukey Test HSD)
