# Supplementary material for: Bioaugmentation of PAH-Contaminated Soils With Novel Specific Degrader Strains Isolated From a Contaminated Industrial Site. Effect of Hydroxypropyl-β-Cyclodextrin as PAH Bioavailability Enhancer
Source: Front Microbiol. 2019 Nov 14;10:2588. doi: 10.3389/fmicb.2019.02588 (PMC6874150; doi:10.3389/fmicb.2019.02588)
Supplement: Supplementary file 3 [file Table_2.docx]

**TABLE S2.** Parameters obtained from PYR mineralisation in TM soil (TM), TM soil after aging (TME), TM soil inoculated with AX 2BC8 (TM inoculated), TM soil inoculated after aging (TME inoculated), TM soil treated with HPBCD after aging (TME + HPBCD), and TM soil inoculated and treated with HPBCD after aging (TME inoculated +HPBCD). Standard deviation in parenthesis (n = 3). ANOVA GLM.

|  | **Acclimation period (days)** | **Extent of mineralization (%)** | **Mineralisation rate (% day^-1^)** |
| --- | --- | --- | --- |
| TM | 49.0 (3.5) b | 46.1 (3.1) d | 1.43 (0.10) cd |
| TME | 79.2 (4.0) c | 35.1 (3.1) c | 1.20 (0.14) bc |
| TM inoculated | 32.1 (4.9) a | 60.5 (1.5) e | 1.66 (0.27) d |
| TME inoculated | 45.3 (2.7) b | 27.3 (1.7) ab | 0.58 (0.06) a |
| TME + HPBCD | 54.4 (2.9) b | 33.9 (2.9) bc | 1.00 (0.11) b |
| TME inoculated + HPBCD | 46.0 (4.4) b | 25.0 (1.5) a | 0.86 (0.08) ab |
| ANOVA TWO WAYS *  p-value |  |  |  |
| Effect of Inoculum (I) | 0,000 | 0,045 | 0,072 |
| Effect of Aging (A) | 0,000 | 0,000 | 0,000 |
| Interaction (I x A) | 0,005 | 0,000 | 0,002 |
| ANOVA TWO WAYS *  p-value |  |  |  |
| Effect of Inoculum (I) | 0,000 | 0,000 | 0,000 |
| Effect of HPBCD (H) | 0,000 | 0,247 | 0,517 |
| Interaction (I x H) | 0,000 | 0,712 | 0,004 |
| ANOVA ONE WAY ** |  |  |  |
| Effect of (I x A x H) | 0,000 | 0,002 | 0,000 |

* Homogeneity of variance by Levene test (p > 0.05), ANOVA test LSD.

** The same lower case letter indicates no statistically significant differences of means (Tukey Test HSD)
